# Identification of Caprine *KRTAP28-1* and Its Effect on Cashmere Fiber Diameter

**DOI:** 10.3390/genes11020121

**Published:** 2020-01-22

**Authors:** Jiqing Wang, Huitong Zhou, Jon G. H. Hickford, Mengli Zhao, Hua Gong, Zhiyun Hao, Jiyuan Shen, Jiang Hu, Xiu Liu, Shaobin Li, Yuzhu Luo

**Affiliations:** 1 Gansu Key Laboratory of Herbivorous Animal Biotechnology, Faculty of Animal Science and Technology, Gansu Agricultural University, Lanzhou 730070, China; wangjq@gsau.edu.cn (J.W.); huitong.zhou@lincoln.ac.nz (H.Z.); Jon.hickford@lincoln.ac.nz (J.G.H.H.); 18394187234@163.com (M.Z.); hua.gong@lincoln.ac.nz (H.G.); haozy2018@163.com (Z.H.); shenjy@st.gsau.edu.cn (J.S.); huj@gsau.edu.cn (J.H.); liuxiu@gsau.edu.cn (X.L.); lisb@gsau.edu.cn (S.L.); 2 International Wool Research Institute, Faculty of Animal Science and Technology, Gansu Agricultural University, Lanzhou 730070, China; 3 Gene-Marker Laboratory, Faculty of Agriculture and Life Sciences, Lincoln University, Lincoln 7647, New Zealand

**Keywords:** goat, keratin-associated protein 28-1 gene (*KRTAP28-1*), variation, cashmere fiber diameter

## Abstract

The keratin-associated proteins (KAPs) are constituents of cashmere fibers and variation in many KAP genes (*KRTAPs*) has been found to be associated with fiber traits. The gene encoding the high-sulphur KAP28-1 has been described in sheep, but it has not been identified in the goat genome. In this study, a 255-bp open reading frame on goat chromosome 1 was identified using a search of similar sequence to ovine *KRTAP28-1*, and that would if transcribed and translated encode a high sulphur KAP. Based on the analysis of polymerase chain reaction amplicons for the goat nucleotide sequences in 385 Longdong cashmere goats in China, five unique banding patterns were detected using single strand conformation polymorphism analysis. These represented five DNA sequences (named variants *A* to *E*) and they had the highest resemblance to *KRTAP28-1* sequences from sheep, suggesting *A*–*E* are variants of caprine *KRTAP28-1*. DNA sequencing revealed a 2 or 4-bp deletion and eleven nucleotide sequence differences, including four non-synonymous substitutions. Of the four common variants (*A*, *B*, *C* and *D*) found in these goats, the presence of variant *A* was associated with decreased mean fiber diameter and this effect appeared to be additive. These results indicate that caprine *KRTAP28-1* variation might have value as a molecular marker for reducing cashmere mean fiber diameter.

## 1. Introduction

Cashmere fiber is produced by the secondary fiber follicles of cashmere goats. As a consequence of its physical properties, the price of cashmere is generally much higher than that of wool. China is the largest producer of cashmere fiber, producing a third to a half of total global cashmere production, and this is widely exported. The Gansu province of China is the main region of cashmere goat breeding in China, and it is home to well-known cashmere goat breeds including the Hexi cashmere goat, the Longdong cashmere goat and the Zhongwei goat [1].

The Longdong cashmere goat is a breed that has been created as a cross between the Liaoning cashmere goat, the Inner Mongolian cashmere goat and the Ziwuling black goat. It is used for both cashmere and meat production and is well adapted to harsh environments including desert and other arid regions. The number of Longdong cashmere goats in China is approximately 1.6 million [1]. Compared to other cashmere goat breeds, the Longdong cashmere goat has a lower mean fiber diameter, but also has a reduced fleece weight [1].

Cashmere fibers are proteinaceous and consist of keratins and keratin-associated proteins (KAPs). The former is assembled into intermediate filaments (IFs), while the latter cross-link the keratins in the IFs to form a semi-rigid matrix. It is believed that both types of protein play important roles in governing the properties of the fibers [2].

The KAPs are proteins that typically have either a high content of cysteine, or of both glycine and tyrosine [2]. According to their amino acid composition, mammalian KAPs have historically been distributed into three broad groups: the high glycine/tyrosine (HGT; 35−60 mol% glycine and tyrosine), the high sulphur (HS; ≤30 mol% cysteine) and the ultra-high sulphur (UHS; >30 mol% cysteine) KAPs [2]. They have been further subdivided into KAP families.

Compared to humans and sheep, the number of KAP genes (*KRTAPs)* identified to date in the goat genome is relatively small. For example, at least 80 functional *KRTAP*s from 25 families and 36 *KRTAP*s from 19 families have been reported in humans [3,4] and sheep [2,5,6,7,8,9,10] respectively. However, only 14 *KRTAP*s from 10 families have been identified in goats [11], with this suggesting that many of the KAP genes found in humans and sheep, remain to be identified. Of the *KAP* genes identified in goats, variation in five *KRTAPs* (*KRTAP8-2*, *KRTAP13-1*, *KRTAP20-1*, *KRTAP20-2* and *KRTAP24-1*) has been found to affect cashmere traits including mean fiber diameter, cashmere weight and crimped fiber length [11,12,13,14,15].

The *KRTAP28-1* gene, which encodes for a high sulphur KAP protein, has been described in sheep [6]. Six variants of the gene, containing eight sequence variations have been reported, and associations between ovine *KRTAP28-1* genotype and mean fiber diameter have been found [6]. This suggests that *KRTAP28-1* is polymorphic and that the effect of variation in *KRTAP28-1* on fiber traits is worthy of investigation in other species. However, KAP28-1 (or its encoding gene *KRTAP28-1*) has been not identified in goats up to now. In this study, we describe the identification of caprine *KRTAP28-1* and the detection of nucleotide sequence variation in this gene. We used a polymerase chain reaction-single strand conformation polymorphism (PCR-SSCP) approach, as this technique enables the rapid and cost-effective screening of large numbers of amplicons for the detection of nucleotide sequence variation, which can then be confirmed by DNA sequencing. We also investigate the effect of variation in the gene on cashmere fiber traits in Longdong cashmere goats. 

## 2. Materials and Methods 

### 2.1. Goats Investigated and Cashmere Data Collection

The animal work was approved by Gansu Agricultural University, and all experiments for these goats were conducted according to the guidelines for the care and use of experimental animals established by the Ministry of Science and Technology of the People’s Republic of China (Approval number 2006-398).

A total of 385 Longdong cashmere goats were studied. These were farmed by the Yusheng Cashmere Goat Breeding Company, and located in Huan County of the Gansu Province of China. These goats were the progeny of eleven un-related sires. At one year of age (first combing to collect fiber), the weight of cashmere fiber obtained by combing was measured. Fiber samples were collected from the mid-side region to measure both mean fiber diameter and the crimped fiber length per goat using an Optical-based Fiber Length and Diameter Analyzer OFDA4000 (EPCO, Shanghai, CHN), which standard deviation is less than 0.07 μm for the machine. Blood samples from these goats were collected onto Munktell TFN paper (Munktell Filter AB, Falun, Sweden) and the DNA from the blood was purified for PCR-SSCP analysis by the method of Zhou et al. [16].

### 2.2. Search for the Caprine KAP28-1 Gene

Using the ovine *KRTAP28-1* sequence [6], a BLASTN search in GenBank of the Caprine Genome Assembly GCF_001704415.1 was undertaken. Of all the sequences found by the BLASTN search, the sequence with the greatest similarity to the ovine *KRTAP28-1* sequence, was assumed to be caprine *KRTAP28-1*.

### 2.3. Polymerase Chain Reaction-Single Strand Conformation Polymorphism (PCR-SSCP) Analysis of Caprine KRTAP28-1

Using the goat sequence identified above, two primers (5′-TAGACAAGCCATTCTCTGTTG-3′ and 5′-CATTCCAGTATTCCTGCCTG-3′) were designed to amplify a 525-bp fragment containing the whole coding region of the putative caprine *KRTAP28-1*. These primers were synthesized by the Takara Biotechnology Company Limited (Dalian, China). Amplifications were carried out in a 20-μL reaction consisting of 0.25 μM of each primer, the genomic DNA on one 1.2-mm punch of TFN paper, 0.5 U of Taq DNA polymerase (Takara, Dalian, China), 150 μM of each dNTP (Takara), 2.5 mM Mg^2+^, 2.0 μL of 10× PCR buffer (Supplied with the DNA polymerase enzyme) and deionized water to make up the volume to 20 μL. The thermal profile consisted of an initial denaturation for 2 minutes at 94 °C, followed by 35 cycles of 94 °C for 30 seconds, 58 °C for 30 seconds and 72 °C for 30 seconds, with a final extension of 5 minutes at 72 °C. The PCR amplifications were carried out in Bio-Rad S1000 thermal cyclers (Bio-Rad, Hercules, CA, USA).

For PCR-SSCP analysis, a 0.7-μL aliquot of each amplicon was mixed with 7 μL of loading dye (0.025% bromophenol blue, 98% formamide, 0.025% xylene-cyanol and 10 mM EDTA). These samples were denatured at 95 °C for 5 minutes, followed by rapidly being cooled on wet ice. They were then immediately loaded onto 16 cm × 18 cm, 12% acrylamide:bisacrylamide (37.5:1; Bio-Rad) gels. Electrophoresis was performed for 20 hours in 0.5× TBE at 300 V and 17.5 °C using Protean II xi cells (Bio-Rad) and the gels were silver-stained based using the method of Byun et al. [17].

### 2.4. Sequencing of KRTAP28-1 Variants and Sequence Analyses

The DNA sequencing approaches employed were different for goats that were homozygous versus heterozygous for particular PCR-SSCP patterns.

Those amplicons that were identified as homozygous by PCR-SSCP analysis, were directly sequenced in both directions using a Sanger sequencing approach at the Beijing Genomics Institute, Beijing, China. However, for those variants that were only present in goats that were identified as being heterozygous, samples were sequenced using the approach described by Gong et al. [18].

DNAMAN version 5.2.10 (Lynnon BioSoft, Vaudreuil, Canada) was used to align DNA and amino acid sequences, and to translate DNA. MEGA version 7.0 was used to construct maximum parsimony phylogenetic tree based on the predicted amino acid sequences. The numbering of nucleotides and amino acids were in accordance with the guidelines at HGVS nomenclature and goat KAP gene sequences were obtained from GenBank and Caprine Genome Assembly GCF_001704415.1.

### 2.5. Statistical Analyses

IBM SPSS Statistics version 24.0 (IBM, NY, USA) was used to perform the statistical analyses. For the common variants (with a frequency greater than 5%), general linear mixed-effects models (GLMMs) were used to assess the effect of the presence or absence (coded as 1 or 0 respectively) of these *KRTAP28-1* variants on various cashmere traits in the 385 Longdong cashmere goats. Since sire and gender were found to affect all the cashmere fiber traits, they were fitted as a random and fixed factor, respectively. Birth rank was not found to affect any cashmere fiber traits, and accordingly it was not included in the models. Only the main effects were tested. Unless otherwise indicated, all *p* values were considered significant when *p* < 0.05.

A second set of analyses was performed with the number of variant copies present included (in place of presence/absence) to ascertain whether additive, dominant or recessive effects were present. These models were conducted in an identical manner to the GLMMs used for testing the presence/absence of each variant.

## 3. Results

### 3.1. Identification of KRTAP28-1 in the Goat Genome

**Figure 1:**
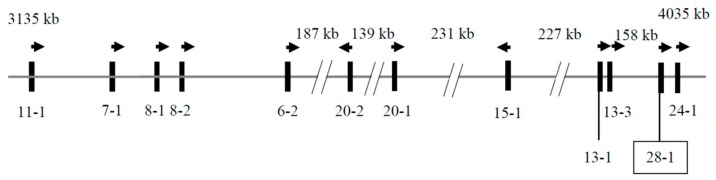
Location of *KRTAPs* on caprine chromosome 1. The identified *KRTAP28-1* sequence is shown in the box, together with eleven previously identified *KRTAPs*. The vertical bars represent the *KAP* genes and the names of these genes are identified below the bars (e.g., 28-1 represents *KRTAP28-1*). The arrows indicate the direction of transcription. The spacing of these genes is only approximate and is based on the Caprine Genome Assembly, as are the nucleotide coordinates.

A BLASTN search of the caprine Genome Assembly GCF_001704415.1 using a sheep *KRTAP28-1* sequence [6] revealed a region on goat chromosome 1 that had 95.07% identity with ovine *KRTAP28-1*. Sequence analysis in the region resulted in the identification of a 255-bp open reading frame (ORF; NC_030808.1: nt4027258_4027512). The ORF was clustered with eleven previously identified *KRTAPs* on goat chromosome 1, with *KRTAP11-1*, *KRTAP7-1*, *KRTAP8-1*, *KRTAP8-2*, *KRTAP6-2*, *KRTAP20-2, KRTAP20-1*, *KRTAP15-1*, *KRTAP13-1* and *KRTAP13-3* being positioned upstream, and only *KRTAP24-1* being located downstream (Figure 1).

**Figure 2:**
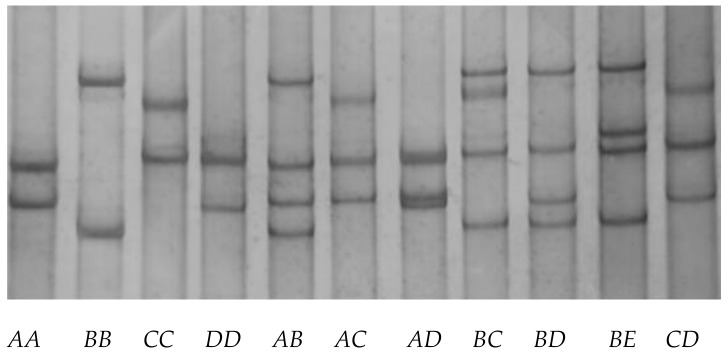
Representative PCR-SSCP patterns of the caprine *KAP28-1* gene. Five unique banding patterns representing five variants (*A*–*E*) were shown in either homozygous or heterozygous forms.

Five different banding patterns (*A*, *B*, *C*, *D* and *E*) were detected in the Longdong cashmere goats by PCR-SSCP analysis (Figure 2). Either one, or a combination of two different patterns, was observed for each goat, which is in accordance with them being either homozygous or heterozygous. DNA sequencing of the amplicons producing these patterns confirmed the occurrence of five unique nucleotide sequences for the amplicons. While all of the five sequences were different, they had over 97% similarity to the DNA sequence in the caprine genome assembly GCF_001704415.1.

**Figure 3:**
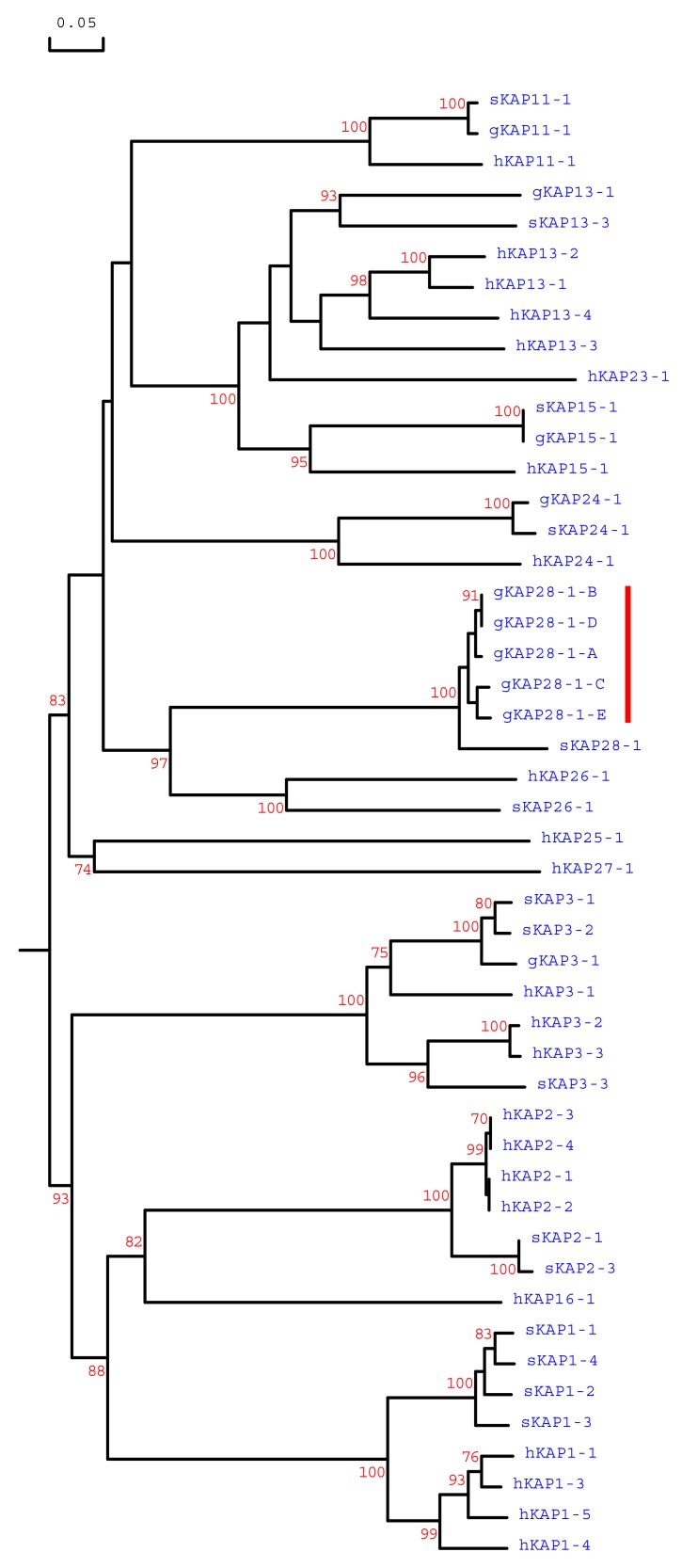
Maximum parsimony tree of the high sulphur KAPs identified in goats, sheep and humans. The tree was constructed using the amino acid sequences (or predicted amino acid sequences). The numbers at the forks indicate the bootstrap confidence values, and only those equal to, or higher than 70%, are shown. The caprine, sheep and human KAPs are indicated with a prefix “g”, ‘s” and “h”, respectively. The five newly identified goat KAP28-1 sequences are indicated with a red vertical line, and the GenBank/EMBL accession numbers for other HS-KAPs are: NM_001285774 (gKAP3-1), NM_001285767.1 (gKAP11-1), AY510115 (gKAP13-1), MG996011 (gKAP24-1), X01610 (sKAP1-1 and sKAP1-4), HQ897973 (sKAP1-2), X02925 (sKAP1-3), P02443 (sKAP2-1), P02441 (sKAP2-3), P02446 (sKAP3-1), P02444 (sKAP3-2), P02445 (sKAP3-3), HQ595347 (sKAP11-1), JN377429 (sKAP13-3), KX817979 (sKAP15-1), JX112014 (sKAP24-1), KX644903 (sKAP26-1), MN053915 (sKAP28-1), NM_030967.2 (hKAP1-1), NM_030966.1 (hKAP1-3), NM_001257305.1 (hKAP1-4), NM_031957.1 (hKAP1-5), NM_001123387.1 (hKAP2-1), NM_033032.2 (hKAP2-2), NM_001165252.1 (hKAP2-3), NM_033184.3 (hKAP2-4), NM_031958.1 (hKAP3-1), NM_031959.2 (hKAP3-2), NM_033185.2 (hKAP3-3), NM_175858.2 (hKAP11-1), NM_181599.2 (hKAP13-1), NM_181621.3 (hKAP13-2), NM_181622.1 (hKAP13-3), NM_181600.1 (hKAP13-4), NM_181623.1 (hKAP15-1), NM_001146182.1 (hKAP16-1), NM_181624.1 (hKAP23-1), NM_001085455.2 (hKAP24-1), NM_001128598.1 (hKAP25-1), NM_203405.1 (hKAP26-1) and NM_001077711.1 (hKAP27-1).

Phylogenetic analysis of the predicted amino acids sequences of the five caprine sequences identified, with all of the high sulphur KAP genes identified in sheep, humans and goats to date, and including the *KRTAP28-1* sequence from sheep, revealed that these caprine sequences was different from all known caprine high sulphur KAP genes, but were most closely related to the ovine *KRTAP28-1* sequence (Figure 3). This suggests that the five sequences identified in the study represent caprine orthologous variants of *KRTAP28-1*.

The five caprine *KRTAP28-1* sequences would all encode polypeptides of 84 amino acid residues. These included high levels of serine (19.05%) and threonine (13.10%), and moderate levels of tyrosine (7.14%), asparagine (7.14%), asparagine (7.14%), phenylalanine (5.95%–7.14%), glycine (4.76%–5.95%), leucine (4.76%–5.95%), cysteine (4.76%) and arginine (3.57%–4.76%).

### 3.2. Detection of Sequence Variation in Caprine KRTAP28-1

**Figure 4:**
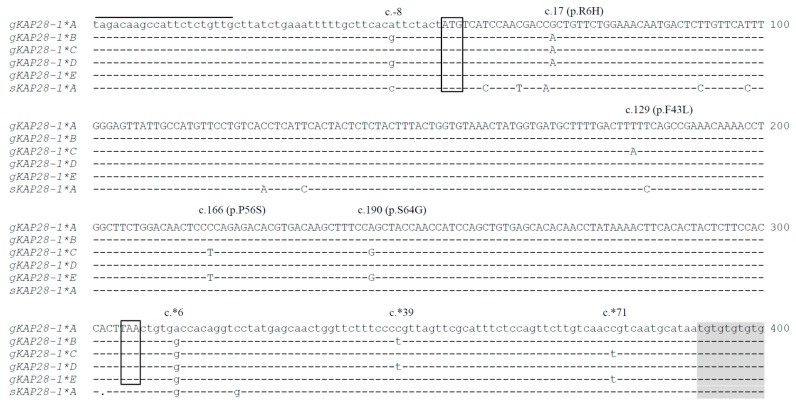

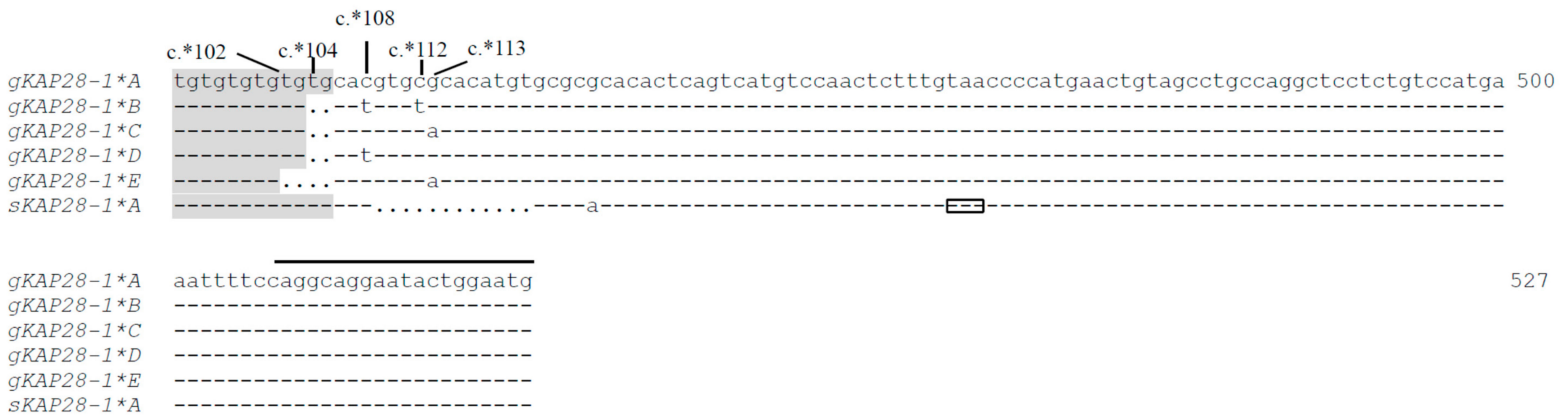
Sequence alignment of caprine *KRTAP28-1* variants *A*-*E* along with ovine *KRTAP28-1* variant *A* (Bai et al., 2019). The primer binding regions are designated with horizontal lines. A prefix “g” and “s” represents sequences from goat and sheep, respectively. Dashes indicate nucleotide identical to the caprine *KRTAP28-1 A* variant sequence, while dots have been introduced to improve the alignment. Nucleotides in the coding region of caprine *KRTAP28-1* are shown in uppercase, while those outside the coding region of caprine *KRTAP28-1* are in lowercase. Eleven nucleotide substitutions and a 2 (or 4)-bp deletion identified in caprine *KRTAP28-1* are marked above the sequences, with the non-synonymous substitutions also indicated. The putative start codon (ATG) and stop codons (TAA) are boxed. The numbering of nucleotides and amino acids are in accordance with the guidelines at HGVS nomenclature (http://varnomen.hgvs.org/). The TG dinucleotide non-coding repeat region is shaded.

Eleven nucleotide variations were found among the five sequences. Of these, one (c.-8A/G) was located in the 5’ untranslated region (UTR), six (c.*6A/G, c.*39C/T, c.*71C/T, c.*108C/T, c.*112C/T and c.*113G/A) were located in the 3’UTR, and the remaining variations (c.17G/A, c.129T/A, c.166C/T and c.190A/G) were within the coding region. It is notable that all sequence variations in the coding region were non-synonymous and would result in a putative amino acid change of p.Arg6His, p.Phe43Leu, p.Pro56Ser and p.Ser64Gly, respectively (Figure 4). When compared to the sequence of *A*, variants *B*, *C* and *D* had a 2-bp deletion (c.*104_*105delTG), but variant *E* had a 4-bp deletion (c.*102_*105delTGTG). This underpinned variation in the number of TG dinucleotide repeats between the variants, with 11 TG repeats in variant *A*, 10 TG repeats in variants *B*, *C* and *D*, and nine TG repeats in variant *E* (Figure 4). In the regions around c.-8, c*39 and c*108, variants *A*, *C* and *E* were identical, and *B* and *D* were identical. In the region spanning c.166 to c.190 and in the region around c.*71 and c.*113, *A*, *B* and *D* were identical, while *C* and *E* were identical. In contrast, in the regions around c.17, *B*, *C* and *D* were identical, while *A* and *E* were identical. In the regions around c.129, c.*6 and c.*112, only one sequence was different in the five nucleotide sequences identified in the study (Figure 4).

### 3.3. Variant and Genotype Frequencies of KRTAP28-1 in the Longdong Cashmere Goats 

In the 385 Longdong cashmere goats investigated, *A* was the most common variant with a frequency of 40.52%, followed by *B*, *C*, *D* and *E* with a frequencies of 24.03%, 19.35%, 14.03% and 2.07%, respectively. Eleven genotypes were observed and there were *AA* (20.52%), *AB* (16.62%), *BB* (6.75%), *AC* (16.10%), *BC* (7.27%), *CC* (4.16%), *AD* (7.27%), *BD* (6.49%), *CD* (7.01%), *DD* (3.65%) and *BE* (4.16%).

### 3.4. Effect of Variation in KRTAP28-1 on Cashmere Traits 

Of the five variants found in the cashmere goats, variant *E* was present at a frequency of less than 5%. It was therefore excluded from the association analyses given the potential for bias. Associations were accordingly only investigated for the four common variants (*A*, *B*, *C* and *D*).

The presence of variant *A* was found to be associated with decreased mean fiber diameter (present: 13.5 ± 0.03 μm; absent: 13.7 ± 0.04 μm; *p* = 0.001). The presence of variant *B* tended to be associated with increased mean fiber diameter (present: 13.6 ± 0.04 μm; absent: 13.5 ± 0.03 μm; *p* = 0.05) and the presence of variant *C* also tended to be associated with increased mean fiber diameter (present: 13.6 ± 0.04 μm; absent: 13.5 ± 0.03 μm; *p* = 0.073). The presence of variant *A* had a tendency to decrease crimped fiber length (present: 4.2 ± 0.04 cm; absent: 4.3 ± 0.04 cm; *p* = 0.084). No association was found between *KRTAP28-1* variants and cashmere fleece weight in these goats (Table 1).

Cashmere fibers from goats with two copies of *A* had lower mean fiber diameter than those from goats with one copy of *A*, whereas cashmere fibers from goat with one copy of *A* had lower mean fiber diameter than those from goats that did not contain *A* (Table 2). This suggested an additive effect of *KRTAP28-1* variation on mean fiber diameter.

## 4. Discussion

Together with homology searching, PCR-SSCP has been proved to be a useful method for finding and characterizing caprine KAP genes, including *KRTAP15-1* [19], *KRTAP20-1* [15], *KRTAP20-2* [14] and *KRTAP24-1* [11]. In this study, the identification of a new high sulphur KAP (called *KRTAP28-1*) has been described. The gene was clustered with eleven previously identified KAP genes on goat chromosome 1 and displayed the highest similarity with the *KRTAP28-1* from sheep when compared to other high sulphur KAP sequences that have been identified in humans, sheep and goats. The identification of *KRTAP28-1* brings the total number of caprine *KRTAPs* described in the published literature, from 14 to 15. 

While the putative polypeptide encoded by the notional caprine *KRTAP28-1* could be classified into the high sulphur KAP group, the content of cysteine (4.76 mol%) in the putative protein was the lower than previously identified caprine high sulphur KAP proteins. In contrast, the caprine KAP28-1 would have a higher content of serine (19.05%), threonine (13.10%) and tyrosine (7.14%). The variation in amino acid composition has been described previously for caprine KAP15-1 [19] and KAP24-1 [11], although its biological implications and function is unknown. While it has been reported that cysteine can form disulfide bond cross-links with the IFs [20], tyrosine in HGT-KAPs are thought to regulate the arrangements of IFs by cation-π interactions [21]. Furthermore serine, threonine and tyrosine have been suggested to be phosphorylated in caprine KAP20-2 [14] and KAP20-1 [15], and phosphorylation has been reported to affect keratin assembly and organization [22]. Accordingly, the functional significance of the higher content of serine, threonine and tyrosine in caprine KAP28-1 deserves further study.

Despite the predicted amino acid sequences of caprine *KRTAP28-1* having the highest similarity to ovine *KRTAP28-1*, there are some differences in the sequences between the two species. First, when compared to goat sequences, there was a 1-bp deletion (c.249_251delA) in the sheep sequence. The deletion would cause the loss of a stop codon and this putatively leads to a 47 amino acid increase in the length of the sheep protein compared to goat KAP28-1. Second, there is variability in the number of TG dinucleotide repeats in the putative KAP28-1 sequences. The sheep sequence includes eleven repeats of TG, while the goat KAP28-1 sequences have nine to eleven repeats. The third difference is the composition of amino acid in KAP28-1. For example, the putative caprine proteins contain 4.76 mol% cysteine, which is lower than that reported for sheep (8.40 mol%). In contrast, the goat sequences have a higher content of serine (19.05 mol%) and tyrosine (13.10 mol%) than sheep (18.61 mol% and 5.46 mol%, respectively). It was further found that compared to goat sequences, more cysteine residues and less serine and tyrosine residues in sheep are mainly arisen from additional length of the ovine KAP28-1 sequences as there was an average levels of 15.55%, 6.68% and 2.00% for cysteine, serine and tyrosine in these additional ovine sequences, respectively.

Sequence variation in *KRTAP28-1* has been studied in sheep [6]. Despite the observation that the KAP28-1 gene is polymorphic in both sheep and goats, the nature of the sequence variation detected in the two species appears to be different. With sheep, the vast majority of variation in sequence is located in the coding regions except for c.-30T/G [6]. In contrast, nucleotide sequence variations in the non-coding regions were found to predominate in goats. This suggests that the polymorphism observed in these two species may be derived by different mechanisms. It is also notable that for both species, all of the coding region sequence variation was non-synonymous. 

Of the eleven sequence variations detected in the study, c.17G/A, c.129T/A, c.166C/T and c.190A/G were non-synonymous, and would lead to amino acid changes in the putative KAP24-1 protein. The substitution c.129T/A would result in the gain or loss of phenylalanine. Due to the possession of an aromatic side chain, phenylalanine has the ability to form a stacking interaction with other residues with aromatic side chains [23]. It is therefore possible that the gain of phenylalanine may contribute to interactions between KAP28-1 and the IFs, and thus affect fiber traits. The substitution c.166C/T could result in change in the mol % content of serine and the substitution c.190A/G could result in the gain or loss of serine and glycine. The absence or presence of serine and glycine may affect KAP structure, as these amino acids can affect helix formation, and thus potentially affect KAP interaction with the IFs. Although nucleotide sequence changes in the non-coding region would not result in amino acid changes, their potential function should not be ignored because they may be linked to other variation in critically important regions of caprine *KRTAP28-1*, and potentially affect transcription binding sites, transcription efficiency and mRNA stability, thereby resulting in altered function [24]. It should be noted that the coding sequence of variant *B* was identical to *D*, and thus if expressed the two variants would arguably produce an identical amino acid sequence.

Of the three cashmere fiber traits investigated in the study, variation in *KRTAP28-1* was only associated with mean fiber diameter, but not cashmere weight and crimped fiber length. The result was in accordance with what was observed in sheep, where *KRTAP28-1* genotype was found to have an effect on mean fiber diameter in Southdown × Merino-cross lambs [6]. The observation that variant *A* was the most common variant in the 385 Longdong cashmere goats, appears to be consistent with the association analyses results. Given that the presence of *A* was associated with a ‘favorable’ cashmere fiber trait (i.e., decreased mean fiber diameter), it might be concluded that purposeful selection for decreased mean fiber diameter in the Longdong cashmere goats has resulted in variant *A* becoming more common than variants *B*, *C*, *D* and *E*.

It is known that ovine *KRTAPs* are clustered together in specific chromosomal regions. The *KRTAP28-1* sequence identified in the study was clustered with eleven previously identified *KRTAPs* on goat chromosome 1. When compared to the associations between variation in these *KRTAPs* and variation in cashmere fiber traits, the effect of variation in *KRTAP28-1* on cashmere traits is similar to that described for *KRTAP24-1* [11], but is different to that reported for *KRTAP20-1* [15], *KRTAP20-2* [14] and *KRTAP13-1* [13]. Given that the *KAP28-1* gene is positioned closer to *KRTAP24-1* than *KRTAP20-1*, *KRTAP20-2* and *KRTAP13-1* on the goat chromosome 1, it is possible that the effect described here for *KRTAP28-1* may be due to its tight linkage to the *KAP24-1* gene, instead of it having an independent effect.

While identifying genes like *KRTAP28-1* is in itself reasonably straight-forward, it has other important implications. For example, high-throughput RNA sequencing (RNA-seq) technology has been widely used in the analysis of the genetic basis of complex and economically important traits in animals. However, the approach requires genes to have been identified and characterized, such that the RNA sequencing reads can be mapped to the reference genome of specific organisms. If there is incomplete annotation of the reference genome (i.e., the sequences have not been identified), then the approach is of limited value. In this context, while it is widely accepted that the KAPs are structural components of cashmere fibers and thus play a role in determining fiber properties, few of the caprine KAP genes have been identified, compared with humans and sheep. Accordingly, the use of RNA-seq technology is unlikely to be of much use in describing follicle activity and the production of cashmere fiber until all the KAPs and keratins have been identified.

## Figures and Tables

**Table 1:** Association of *KRTAP28-1* variants with various cashmere traits (mean ± SE) ^1^ in Longdong cashmere goats.

Cashmere Trait (unit)	Variant	Absent		Present		*p* Value ^1^
		Mean ± SE	*n*	Mean ± SE	*n*	
Cashmere weight (g)	*A*	411 ± 3.9	152	412 ± 3.4	233	0.730
	*B*	413 ± 3.4	226	409 ± 3.8	159	0.322
	*C*	412 ± 3.3	252	410 ± 4.1	133	0.729
	*D*	411 ± 3.2	291	412 ± 4.8	94	0.843
Mean fiber diameter (μm)	*A*	**13.7 ± 0.04**	**152**	**13.5 ± 0.03**	**233**	**0.001**
	*B*	13.5 ± 0.03	226	13.6 ± 0.04	159	0.050
	*C*	13.5 ± 0.03	252	13.6 ± 0.04	133	0.073
	*D*	13.6 ± 0.03	291	13.6 ± 0.05	94	0.467
Crimped fiber length (cm)	*A*	4.3 ± 0.04	152	4.2 ± 0.04	233	0.084
	*B*	4.2 ± 0.04	226	4.2 ± 0.04	159	0.438
	*C*	4.2 ± 0.03	252	4.2 ± 0.04	133	0.652
	*D*	4.2 ± 0.03	291	4.2 ± 0.05	94	0.951

^1^ Estimated marginal means and standard errors (SE) of those means derived from general linear mixed-effects models that included “gender’ as a fixed factor and “sire” as a random factor. *p* < 0.05 are in bold.

**Table 2:** Association of caprine *KRTAP28-1* variant copy number with various cashmere traits (Mean ± SE) ^1^ in Longdong cashmere goats.

Cashmere Trait (unit)	Variant	Absent		One Copy		Two Copies		*p* Value
		Mean ± SE	*n*	Mean ± SE	*n*	Mean ± SE	*n*	
Cashmere weight (g)	*A*	411 ± 3.9	152	410 ± 3.9	154	417 ± 5.2	79	0.431
	*B*	413 ± 3.4	226	408 ± 4.1	134	411 ± 4.1	25	0.585
	*C*	412 ± 3.3	252	410 ± 4.3	117	411 ± 3.6	16	0.632
	*D*	411 ± 3.2	291	412 ± 5.1	80	410 ± 6.2	14	0.967
Mean fiber diameter (μm)	*A*	**13.7 ± 0.04^Aa^**	**152**	**13.5 ± 0.04^ABb^**	**154**	**13.4 ± 0.05^Bc^**	**79**	**<0.001**
	*B*	13.5 ± 0.03	226	13.6 ± 0.04	134	13.6 ± 0.06	25	0.141
	*C*	13.6 ± 0.03	252	13.6 ± 0.04	117	13.7 ± 0.06	16	0.094
	*D*	13.6 ± 0.03	291	13.6 ± 0.05	80	13.7 ± 0.05	14	0.523
Crimped fiber length (cm)	*A*	4.2 ± 0.04	152	4.2 ± 0.04	154	4.2 ± 0.06	79	0.220
	*B*	4.2 ± 0.04	226	4.2 ± 0.04	134	4.3 ± 0.05	25	0.454
	*C*	4.2 ± 0.03	252	4.2 ± 0.04	117	4.3 ± 0.04	16	0.656
	*D*	4.2 ± 0.03	291	4.2 ± 0.05	80	4.2 ± 0.06	14	0.976

^1^ Estimated marginal means and standard errors (SE) of those means derived from general linear mixed-effects models that included “gender’ as a fixed factor and “sire” as a random factor. Means within rows that do not share a superscript uppercase (A or B) are different at *p* < 0.01, while means within rows that do not share a superscript lowercase (a, b or c) are different at *p* < 0.05. *p* < 0.05 are in bold.
